# Macrocyclic peptides: up-and-coming weapons to combat antimicrobial resistance

**DOI:** 10.1038/s41392-024-01813-4

**Published:** 2024-04-02

**Authors:** Wen-Jing Wang, Xiang-Min Dong, Guo-Bo Li

**Affiliations:** 1 Department of Biotherapy, Cancer Center and State Key Laboratory of Biotherapy, West China Hospital, Sichuan University, Chengdu, Sichuan China; 2 Key Laboratory of Drug Targeting and Drug Delivery System of Ministry of Education, Department of Medicinal Chemistry, West China School of Pharmacy, Sichuan University, Chengdu, Sichuan China

**Keywords:** Microbiology, Infection

Recently, two companion papers published in *Nature* reported a first-in-class tethered macrocyclic peptide antibiotic targeting carbapenem-resistant *A. baumannii* (CRAB) and unveiled their unique antibacterial mechanism, which involves disrupting outer membrane structures by targeting lipopolysaccharide (LPS) transporters.^1,2^

The emergence of antibiotic-resistant pathogens is increasingly recognized as a critical threat to global health. The development of new antibiotics has undergone a prolonged downturn, particularly for *A. baumannii*, where no new antibiotic class has reached patients in over five decades. Among the antibiotic-resistant bacteria identified by the World Health Organization, CRAB holds the top position, which involves evolved serine/metallo β-lactamases and other resistance mechanisms.^3^ In clinical practice, some older antibiotics (such as polymyxins) with safety concerns are being used to treat CRAB infections. The β-lactamase inhibitor durlobactam, in combination with sulbactam, was approved in the United States in May 2023, potentially offering a new treatment avenue for CRAB infections. Estimates for mortality rates associated with invasive CRAB infections range between 40 and 60 percent, underscoring the pressing need for effective and safe treatment options.

**Fig. 1:**
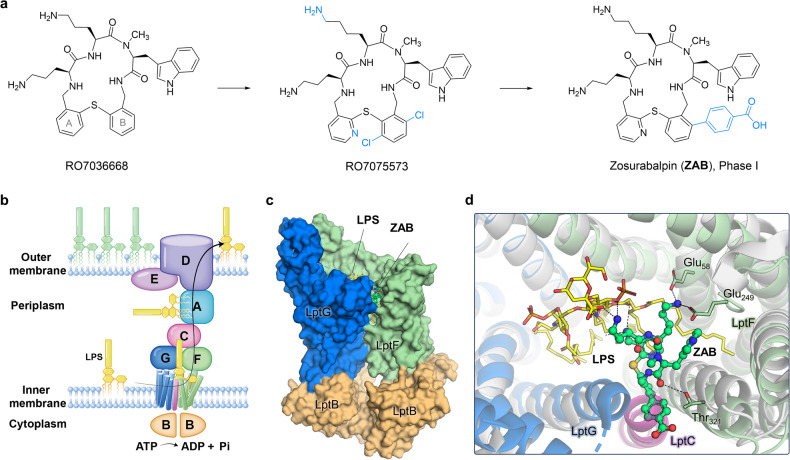
New macrocyclic peptides targeting LPS transporters. **a** Structural optimization led to the drug candidate ZAB; **b** schematic diagram of LptB_2_FGCADE, the seven-protein LPS transport machinery, adapted from the *Nature* study^2^. **c** Structural basis for the *A. baylyi* LptB_2_FG:LPS:ZAB complex (PDB code 8FRN)^2^. **d** Superimposition of the *A. baylyi* LptB_2_FG:LPS:ZAB and LptB_2_FGC (PDB code 8FRP)^2^

Macrocyclic peptides (MCPs) are important sources for discovering new antibiotics due to their constrained conformations and chemical stability. Many clinically important antibiotics are MCPs, such as polymyxins, bacitracin, and daptomycin, which generally exhibit unique and narrow-spectrum antibacterial activity. Zampaloni et al. identified an unprecedented MCP, RO7036668, effective against *A. baumannii* through whole-cell phenotype screening of over 44,000 macrocyclic compounds.^1^ This compound is a seventeen-membered macrocyclic ring composed of an L-Orn-L-Orn-L-*N*(Me)-Trp subunit and a di-o-tolylsulfane moiety (Fig. 1a). It shows activity against *A. baumannii* with a minimum inhibitory concentration (MIC) of 4 μg/ml, but has limited activity against other Gram-negative bacteria and Gram-positive bacteria. The hit-to-lead optimization through replacement of the central L-Orn with L-Lys, replacement of the benzene ring A with pyridine, and dichloro substitution at the benzene ring B resulted in the production of RO7075573 (Fig. 1a). This compound had substantially improved activity, with MICs ranging from ≤0.06 to 0.5 μg/ml against a panel of *A. baumannii* strains, including multidrug-resistant strains. Despite its promising in vivo antibacterial effects and ADMET properties, RO7075573 suffered poor plasma compatibility, leading to the aggregation of low-density lipoprotein/high-density lipoprotein vesicles. Further optimization of physico-chemical properties, by analogizing with standard-of-care antibiotics (e.g., polymyxins and β-lactams), resulted in the discovery of the zwitterionic derivative zosurabalpin (ZAB). ZAB exhibited potent in vitro activity against the challenging and multidrug-resistant human clinical isolates of *A. baumannii*, along with significantly reduced plasma precipitation. This compound possesses favorable preclinical pharmacokinetic and toxicity profiles, and in vivo studies have showcased its efficacy across several mouse infection models. ZAB is currently undergoing evaluation in human clinical trials. The discovery of ZAB, inspired by standard-of-care antibiotics, could be a useful reference for the optimization process, especially in developing clinically useful drugs.

Given that MCPs represent unexplored macrocyclic antibiotics with potent and narrow-spectrum activity against *A. baumannii*, Zampaloni et al. firmly believe that MCPs target a novel mechanism. Eventually, they identified the inner-membrane LptB_2_FGC complex as the target of MCPs through whole-genome sequencing of ZAB-resistant single colonies, along with biochemical assays. In Gram-negative bacteria, the translocation of LPS from the inner to the outer membrane relies on the seven-protein transport machine, LptB_2_FGCADE, which forms a bridge connecting the inner and the outer membrane (Fig. 1b). The inner-membrane components LptB_2_FGC hydrolyzes ATP to provide the necessary energy for LPS transport by LptB_2_FGCADE. LptC receives the LPS transported by LptF and LptG, delivering it to the periplasmic protein LptA. Subsequently, LPS is transferred from the periplasm to LptDE, which inserts LPS into the outer leaflet of the outer membrane (Fig. 1b). Pahil et al. solved high-resolution structures of *A. baylyi* LptB_2_FG with MCPs using cryo-electron microscopy. The structures revealed that MCPs bind within LptB_2_FG alongside LPS. Take ZAB for example, it is positioned amidst several transmembrane helices of LptF and LptG (TM1_LptG_, TM2_LptF_, TM4_LptF_, and TM5_LptF_). Upon binding, ZAB extends from the lateral gate of LptB_2_FG (surface gaps exist between TM1_LptG_ and TM5_LptF_) (Fig. 1c). ZAB is positioned to form hydrogen-bonding interactions with Thr_321_ on LptF, and make electrostatic interactions with Glu_58_ and Glu_249_ on LptF by the central L-Lys, explaining the structure-activity relationships of this series of MCPs. It was observed that in the absence of MCPs, the overall conformation and interaction of LPS with the transporter remained unchanged, suggesting the LPS-loaded transporter could serve as a druggable conformation for antibiotic discovery. Superimposition of the *A. baylyi* LptB_2_FG:LPS:ZAB and LptB_2_FGC structures reveals that ZAB is positioned to obstruct the binding of LptC, thereby impeding the delivery of LPS to LptC (Fig. 1d). Comparison of *A. baylyi* LptB_2_FG:LPS:ZAB with *E. coli* LptB_2_FG:LPS structure^4,5^ revealed differences in their LptF helices and LPS binding positions, explaining why MCPs have narrow-spectrum activity to *A. baumannii*.

In summary, MCPs are a groundbreaking class of antibiotics that potently and selectively target the *A. baumannii* LPS transporter which has not been explored before. The identified clinical candidate ZAB holds promise in addressing the urgent threat of invasive CRAB infections. However, concerns arise regarding the potential short lifecycle of ZAB due to a singular antibacterial mechanism, as observed with decreased drug sensitivity caused by point mutations. The complex structures not only reveal the atomic-level mechanism of MCPs blocking LPS transporters, but more importantly, provides a structural basis for advancing the optimization of MCPs to discover superior antibiotics against *A. baumannii* or broad-spectrum antibiotics for other carbapenem-resistant bacterial pathogens. Furthermore, these findings suggest that MCPs may server as a source for the discovery of molecular glues to combine bacterial infections or other human diseases.
